# Recursive patterns in online echo chambers

**DOI:** 10.1038/s41598-019-56191-7

**Published:** 2019-12-27

**Authors:** Emanuele Brugnoli, Matteo Cinelli, Walter Quattrociocchi, Antonio Scala

**Affiliations:** 1 Applico Lab, CNR-ISC, Rome, 00185 Italy; 2 Ca’ Foscari University of Venice, Venice, 30123 Italy; 3 LIMS, the London Institute for Mathematical Sciences, London, United Kingdom

**Keywords:** Computational science, Scientific data

## Abstract

Despite their entertainment oriented purpose, social media changed the way users access information, debate, and form their opinions. Recent studies, indeed, showed that users online tend to promote their favored narratives and thus to form polarized groups around a common system of beliefs. Confirmation bias helps to account for users’ decisions about whether to spread content, thus creating informational cascades within identifiable communities. At the same time, aggregation of favored information within those communities reinforces selective exposure and group polarization. Along this path, through a thorough quantitative analysis we approach connectivity patterns of 1.2 M Facebook users engaged with two very conflicting narratives: scientific and conspiracy news. Analyzing such data, we quantitatively investigate the effect of two mechanisms (namely challenge avoidance and reinforcement seeking) behind confirmation bias, one of the major drivers of human behavior in social media. We find that challenge avoidance mechanism triggers the emergence of two distinct and polarized groups of users (i.e., echo chambers) who also tend to be surrounded by friends having similar systems of beliefs. Through a network based approach, we show how the reinforcement seeking mechanism limits the influence of neighbors and primarily drives the selection and diffusion of contents even among like-minded users, thus fostering the formation of highly polarized sub-clusters within the same echo chamber. Finally, we show that polarized users reinforce their preexisting beliefs by leveraging the activity of their like-minded neighbors, and this trend grows with the user engagement suggesting how peer influence acts as a support for reinforcement seeking.

## Introduction

Social media facilitated global communications all over the world, allowing information to spread faster and intensively. These changes led up to the formation of a disintermediated scenario, where contents flow directly from producers to consumers, without the mediation of journalists or experts in the field. Beyond its undoubted benefits, a hyper-connected world can foster confusion about causation, and thus encourage speculation, rumors, and mistrust^1–4^. Since 2013, indeed, the World Economic Forum (WEF) has been placing the global threat of massive digital misinformation at the core of other technological and geopolitical risks, ranging from terrorism, to cyber-attacks, up to the failure of global governance^5^. People are misinformed when they hold beliefs neglecting factual evidence, and misinformation may influence public opinion negatively. Empirical investigations have shown that, in general, people tend to resist facts, holding inaccurate factual beliefs confidently^6^. Moreover, corrections frequently fail to reduce misperceptions^7^ and often act as a backfire effect^8^.

Confirmation bias - i.e., the tendency to seek, select, and interpret information coherently with one’s system of beliefs^9^ - helps, indeed, to account for users’ decisions about whether to promote content^2,10–12^. The action of this cognitive bias may lead to the emergence of homogeneous and polarized communities - i.e., echo-chambers^13–15^, thus facilitating fake news and, more in general, misinformation cascades^3^.

According to^16^, two primary cognitive mechanisms are used to explain why people experience the confirmation bias:

- Challenge avoidance - i.e., the fact that people do not want to find out that they are wrong,
- Reinforcement seeking - i.e., the fact that people want to find out that they are right.

Though the two are strongly related, and though both behaviors resolve around people’s attempt to minimize their cognitive dissonance - i.e., the psychological stress that people experience when they hold two or more contradictory beliefs simultaneously, challenge avoidance and reinforcement seeking are not inherently linked to each other, and they do not have to occur at the same time. This distinction is important because the consequences of challenge avoidance are significantly more harmful to democratic deliberation than those of reinforcement seeking^17^. Additionally, group membership has an interplay with the aforementioned cognitive biases. When individuals belong to a certain group, those outside the group are far less likely to influence them on both easy and hard questions^18^.

In this work, by exploiting the social network of 1.2 M Facebook users engaged with very polarizing contents, we investigate the role of challenge avoidance and reinforcement seeking on the selection and spread of information, and the connection of such cognitive mechanisms with peer influence.

To our aim, with the help of very active debunking groups, we identified all the Italian Facebook pages supporting scientific and conspiracy news, and on a time span of five years (2010–2014) we downloaded all their public posts (with the related lists of likes and comments). On the one hand, conspiracy news simplify causation, reduce the complexity of reality, and are formulated in a way that is able to tolerate a certain level of uncertainty^19–21^. On the other hand, scientific news disseminates scientific advances and exhibits the process of scientific thinking. Notice that we do not focus on the quality of the information but rather on the possibility of verification. Indeed, the main difference between the two is content verifiability. The generators of scientific information and their data, methods, and outcomes are readily identifiable and available. The origins of conspiracy theories are often unknown and their content is strongly disengaged from mainstream society and sharply divergent from recommended practices^8^, e.g., the belief that vaccines cause autism^22^.

Our analyses show how challenge avoidance mechanism triggers the emergence, around the selected narratives, of two well-separated and polarized groups of users who also tend to surround themselves with friends having similar systems of beliefs.

Through a network based approach, we also prove that polarized users span their attention focus on a higher number of pages (and topics) supporting their beliefs (hereafter referred to as *community pages*) as their engagement grows, but they tend to remain confined within groups of very few pages even when the corresponding neighborhoods are active on several news sources. This suggests that the reinforcement seeking mechanism limits the influence of neighbors and primarily drives the selection and the diffusion of contents even among like-minded users, fostering the formation of highly polarized subclusters within the same echo chamber.

Finally, we investigate the effects of the joint action of confirmation bias and peer influence when the latter does not conflict the cognitive mechanisms of challenge avoidance and reinforcement seeking. Namely, we compare the liking activity of polarized users and the liking activity of their part of neighborhood likewise polarized, both with respect to size and time. Our findings reveal that polarized users reinforce their preexisting beliefs by leveraging the activity of their like-minded neighbors. Such a trend grows with the user engagement and suggests how peer influence acts as a support for reinforcement seeking. In such a context, also the positive role played by social influence - e.g., by enabling social learning^23–25^, seems to lose its effectiveness in the effort of smoothing polarization and reducing both the risk and the consequences of misinformation. This makes it even more difficult to design efficient communication strategies to prevent rumors and mistrust. Individual choices more than algorithms^10^ seem to characterize the consumption patterns of users and their friends. Therefore, working towards long-term solutions to polarization and misinformation online cannot be separated from a deep understanding of users’ cognitive determinants behind these mechanisms.

## Methods

### Ethics statement

Approval and informed consent were not needed because the data collection process has been carried out using the Facebook Graph application program interface (API), which is publicly available. For the analysis (according to the specification settings of the API) we only used publicly available data (thus users with privacy restrictions are not included in the dataset). The pages from which we download data are public Facebook entities and can be accessed by anyone. User content contributing to these pages is also public unless the user’s privacy settings specify otherwise, and in that case it is not available to us.

### Data collection

Debate about social issues continues to expand across the Web, and unprecedented social phenomena such as the massive recruitment of people around common interests, ideas, and political visions are emerging. For our analysis, we identified two main categories of pages: conspiracy news – i.e., pages promoting contents neglected by main stream media – and science news. We defined the space of our investigation with the support of diverse Facebook groups that are very active in debunking conspiracy theses. As an additional control, we used the self-description of a page to determine its focus. The resulting dataset is composed by all the pages supporting the two distinct narratives in the Italian Facebook scenario: 39 about conspiracy theories and 33 about science news. For the two sets of pages we download all of the posts (and their respective user interactions) across a 5-y time span (2010–2014). We perform the data collection process by using the Facebook Graph API, which is publicly available and accessible through any personal Facebook user account. The exact breakdown of the data is presented in Table 1. Likes and comments have a different meaning from the user viewpoint. Most of the times, a like stands for a positive feedback to the post and a comment is the way in which online collective debates take form. Comments may contain negative or positive feedbacks with respect to the post.

**Table 1:** Breakdown of Facebook dataset.

	Total	Science	Conspiracy
Pages	72	33	39
Posts	270,629	62,038	208,591
Likes	9,164,781	2,505,399	6,659,382
Comments	1,017,509	180,918	836,591
Likers	1,196,404	332,357	864,047
Commenters	279,972	53,438	226,534

The number of pages, posts, likes and comments for science and conspiracy pages.

### Ego networks

In addition, we collected the ego networks of users who liked at least one post on science or conspiracy pages - i.e., for each user we have collected her list of friends and the links between them (We used publicly available data, so we collected only data for which the users had the corresponding permissions open).

### Preliminaries and definitions

Let ${\mathscr P}$ be the set of all the pages in our collection, and ${{\mathscr P}}_{{\rm{science}}}$ (${{\mathscr P}}_{{\rm{conspir}}}$) be the set of the 33 (39) Facebook pages about science (conspiracy) news. Let *V* be the set of all the 1.2 M users and *E* the edges representing their Facebook friendship connections; these sets define a graph ${G}=(V,E)$. Hence, the graph of likes on a post, ${{G}}^{L}=({V}^{L},{E}^{L})$ is the subgraph of *G* whose users have liked a post. Thus, *V*^*L*^ is the set of users of *V* who have liked at least one post, and we set ${E}^{L}=\{(u,v)\in E;u,v\in {V}^{L}\}$. Following previous works^2,3,26^, we study the polarization of users - i.e., the tendency of users to interact with only a single type of information; in particular, we study the polarization towards science and conspiracy. Formally we define the polarization $\rho (u)\in [\,-\,1,1]$ of user $u\in {V}^{L}$ as the ratio of likes that *u* has performed on conspiracy posts: assuming that *u* has performed *x* and *y* likes on conspiracy and science posts, respectively, we let $\rho (u)=(x-y)/(x+y)$. Thus, a user *u* for whom $\rho (u)=-\,1$ is totally polarized towards science, whereas a user with $\rho (u)=1$ is totally polarized towards conspiracy. Note that we ignore the commenting activity since a comment may be an endorsement, a criticism, or even a response to a previous comment. Furthermore, we define the engagement $\psi (u)$ of a polarized user *u* as her liking activity normalized with respect to the number of likes of the most active user of her community. By defining $\theta (u)$ as the total number of likes that the user *u* has expressed in posts of ${\mathscr{P}}$, notice that the following condition holds: $\psi (u)=\frac{\theta (u)}{{{\rm{\max }}}_{v}\,\theta (v)}$.

The degree of a node (here, user) *u*, *deg*(*u*), is the number of neighbors (here, friends) of *u*. For any user *u*, we consider the partition ${\deg }(u)=|{N}_{c}(u)|+|{N}_{ne}(u)|+|{N}_{np}(u)|+|{N}_{s}(u)|$ where ${N}_{c}(u)({N}_{s}(u))$ denotes the neighborhood of *u* polarized towards conspiracy (science), ${N}_{ne}(u)$ denotes the neighborhood of *u* not engaged with science or conspiracy contents, ${N}_{np}(u)$ denotes the set of not polarized friends of *u* - i.e., friends who liked the same number of contents from science and conspiracy, respectively.

To understand the relationship between pages and user liking activity, we measure the polarization of users with respect to the pages of their own community. For a polarized user (or, more in general, a group of polarized users) *u* with ${\sum }_{i}\,{\theta }_{i}(u)=\theta (u)$ likes, where ${\theta }_{i}(u)$ counts the contents liked by *u* on the *i*^th^ community page ($i=1,\ldots ,N$, where *N* equals the number of community pages), the probability ${{\phi }}_{i}(u)$ that *u* belongs to the *i*^th^ page of the community will then be ${{\phi }}_{i}(u)={\theta }_{i}(u)/\theta (u)$. We can define the localization order parameter *L* as:

$$L(u)=\frac{{({\sum }_{i}{{\phi }}_{i}^{2}(u))}^{2}}{{\sum }_{i}\,{{\phi }}_{i}^{4}(u)}$$

Thus, in the case in which *u* only has likes in one page, $L(u)=1$. If *u*, on the other hand, interacts equally with all the community pages (${{\phi }}_{i}(u)=1/N$) then $L(u)=N$; hence, $L(u)$ counts the community pages where *u* fairly equally distributes her liking activity.

### List of pages

In this section are listed pages of our dataset. Table 2 shows the list of scientific news and Table 3 shows the list of conspiracy pages.

**Table 2:** Scientific news sources.

	Page name	Facebook ID
1	Scientificast.it	129133110517884
2	CICAP	32775139194
3	OggiScienza	106965734432
4	Query	128523133833337
5	Gravità Zero	138484279514358
6	COELUM Astronomia	81631306737
7	MedBunker	246240278737917
8	In Difesa della Sperimentazione Animale	365212740272738
9	Italia Unita per la Scienza	492924810790346
10	Scienza Live	227175397415634
11	La scienza come non l’avete mai vista	230542647135219
12	LIBERASCIENZA	301266998787
13	Scienze Naturali	134760945225
14	Perché vaccino	338627506257240
15	Le Scienze	146489812096483
16	Vera scienza	389493082245
17	Scienza in rete	84645527341
18	Galileo, giornale di scienza e problemi globali	94897729756
19	Scie Chimiche: Informazione Corretta	351626174626
20	Complottismo? No grazie	399888818975
21	INFN - Istituto Nazionale di Fisica Nucleare	45086217578
22	Signoraggio: informazione corretta	279217954594
23	Scetticamente	146529622080908
24	Vivisezione e Sperimentazione Animale, verità e menzogne	548684548518541
25	Medici Senza Frontiere	65737832194
26	Task Force Pandora	273189619499850
27	VaccinarSI	148150648573922
28	Lega Nerd	165086498710
29	Super Quark	47601641660
30	Curiosità Scientifiche	595492993822831
31	Minerva - Associazione di Divulgazione Scientifica	161460900714958
32	Pro-Test Italia	221292424664911
33	Uniti per la Ricerca	132734716745038

List of Facebook pages diffusing main stream scientific news.

**Table 3:** Conspiracy news sources.

	Page name	Facebook ID
34	Scienza di Confine	188189217954979
35	CSSC - Cieli Senza Scie Chimiche	253520844711659
36	STOP ALLE SCIE CHIMICHE	199277020680
37	Vaccini Basta	233426770069342
38	Tanker Enemy	444154468988487
39	SCIE CHIMICHE	68091825232
40	MES Dittatore Europeo	194120424046954
41	Lo sai	126393880733870
42	AmbienteBio	109383485816534
43	Eco(R)esistenza	203737476337348
44	curarsialnaturale	159590407439801
45	La Resistenza	256612957830788
46	Radical Bio	124489267724876
47	Fuori da Matrix	123944574364433
48	Graviola Italia	130541730433071
49	Signoraggio.it	278440415537619
50	Informare Per Resistere	101748583911
51	Sul Nuovo Ordine Mondiale	340262489362734
52	Avvistamenti e Contatti	352513104826417
53	Umani in Divenire	195235103879949
54	Nikola Tesla - il SEGRETO	108255081924
55	Teletrasporto	100774912863
56	PNL e Ipnosi	150500394993159
57	HAARP - controllo climatico	117166361628599
58	Sezione Aurea, Studio di Energia Vibrazionale	113640815379825
59	PER UNA NUOVA MEDICINA	113933508706361
60	PSICOALIMENTARSI E CURARSI NATURALMENTE	119866258041409
61	La nostra ignoranza è la LORO forza.	520400687983468
62	HIV non causa AIDS	121365461259470
63	Sapere è un Dovere	444729718909881
64	V per Verità	223425924337104
65	Genitori veg	211328765641743
66	Operatori di luce	195636673927835
67	Coscienza Nuova	292747470828855
68	Aprite Gli Occhi	145389958854351
69	Neovitruvian	128660840526907
70	CoscienzaSveglia	158362357555710
71	Medicinenon	248246118546060
72	TERRA REAL TIME	208776375809817

List of Facebook pages diffusing conspiracy news.

### Augmented Dickey–Fuller test

An augmented Dickey–Fuller test (ADF) tests the null hypothesis that a unit root is present in a time series^27,28^. The alternative hypothesis is stationarity. If we obtain a p-value less than the threshold value $\bar{\alpha }=0.05$, the null hypothesis is rejected in favor of the alternative one. ADF is an augmented version of the Dickey–Fuller test^29^ for a larger set of time series models. We use this test to investigate the stationarity of the time series given by the number of posts per day published by a community page during its lifetime. The general regression equation which incorporates a constant and a linear trend is used. The number of lags used in the regression corresponds to the upper bound on the rate at which the number of lags should be made to grow with the time series size *T* for the general ARMA(*p*, *q*) setup^30^, and equals *T*^1/3^.

### Cosine similarity

Cosine similarity is a measure of similarity between two non-zero vectors ${\bf{u}}=({u}_{1},\ldots ,{u}_{k})$ and ${\bf{v}}=({v}_{1},\ldots ,{v}_{k})$ of a *k*-dimensional inner product space expressed by the cosine of the angle between them^31^. By means of the Euclidean dot product formula we obtain

$$\cos ({\bf{u}},{\bf{v}})=\frac{{\bf{u}}\cdot {\bf{v}}}{\parallel {\bf{u}}\parallel \parallel {\bf{v}}\parallel }=\frac{{\sum }_{i=1}^{k}\,{u}_{i}{v}_{i}}{\sqrt{{\sum }_{i=1}^{k}\,{u}_{i}^{2}}\sqrt{{\sum }_{i=1}^{k}\,{v}_{i}^{2}}}.$$

We use cosine similarity to evaluate whether a polarized user *u* and the part of her neighborhood with likewise polarization proportionally distribute their liking activity across her preferred community pages. Namely, for any user *u* polarized towards science (conspiracy), denoted with $\{{P}_{{i}_{1}},\ldots ,{P}_{{i}_{k}}\}={{\mathscr P}}_{{\rm{science}}}^{u}\,\,({{\mathscr P}}_{{\rm{conspir}}}^{u})$ the set of *k* science (conspiracy) pages where *u* distributes her liking activity, we compute the cosine between the vectors $({\theta }_{{i}_{1}}(u),\ldots ,{\theta }_{{i}_{k}}(u))$ and $({\theta }_{{i}_{1}}({N}_{s}(u)),\ldots ,{\theta }_{{i}_{k}}({N}_{s}(u)))$, both normalized with respect to the infinity norm. The space of such versors is positive, then the cosine measure outcome is neatly bounded in $[0,1]$: two versors are maximally similar if they are parallel and maximally dissimilar if they are orthogonal.

### Akaike information criterion

The Akaike Information Criterion (AIC)^32–34^ is an asymptotically unbiased estimator of the expected relative Kullback-Leibler distance (K-L)^35^, which represents the amount of information lost when we use model *g* to approximate model *f*:

$$K-L=I(f,g)={\int }^{}\,f(x)\,\log \,(\frac{f(x)}{g(x|\mu )})\,d{x}^{2},$$

where $\mu =({\mu }_{1},\ldots ,{\mu }_{k})$ is the vector of *k* model parameters. The AIC for a given model is a function of its maximized log-likelihood ($\ell$) and *k*:

$${\rm{AIC}}=-\,2\ell +2k.$$

We use the AIC for selecting the optimal lag structure of a Granger causality test.

### Granger causality and peer influence probability

The Granger causality test is a statistical hypothesis test for determining whether one time series is useful in forecasting another^36^. Roughly speaking, a time series *X* is said to Granger-cause (briefly, G-cause) the time series *Y* if the prediction of *Y* is improved when *X* is included in the prediction model of *Y*. Denoted with ${ {\mathcal I} }^{\ast }(\tau )$ the set of all information in the universe up to time $\tau$ and with ${ {\mathcal I} }_{-X}^{\ast }(\tau )$ the same information set except for the values of series *X* up to time $\tau$, we write

$${Y}_{\tau +1}{\mathrel{{\perp\mkern-10mu\perp}}}{ {\mathcal I} }^{\ast }(\tau )|{ {\mathcal I} }_{-X}^{\ast }(\tau )$$

for indicating that *X* does not cause *Y*.

Let $t(u)$ be the time series given by the number of likes expressed by a user *u* polarized towards science on ${{\mathscr P}}_{{\rm{science}}}^{u}$ every day of her lifetime - i.e., the temporal distance between its first and its last like. Let $t({N}_{s}(u))$ be the time series of the number of likes expressed by ${N}_{s}(u)$ on the same pages every day in the same time window. We investigate a causal effect of $t({N}_{s}(u))$ on $t(u)$ by testing the null hypothesis that the former does not Granger-cause the latter:

$${{\mathbb{H}}}_{0}:\,=t{(u)}_{\tau +1}{\mathrel{{\perp\mkern-10mu\perp}}}{ {\mathcal I} }^{\ast }(\tau )|{ {\mathcal I} }_{-t({N}_{s}(u))}^{\ast }(\tau )$$

through a series of F-tests on lagged values of $t(u)$. The alternative hypothesis ${{\mathbb{H}}}_{1}$ is $t({N}_{s}(u))$ G-cause $t(u)$. The number of lags to be included is chosen using AIC. If we obtain a p-value *α*(*u*) less than the threshold value $\bar{\alpha }=0.05$, the null hypothesis ${{\mathbb{H}}}_{0}$ is rejected in favor of ${{\mathbb{H}}}_{1}$. The same analysis is carried out for testing a causal effect of $t({N}_{c}(u))$ on $t(u)$ for any polarized user *u* towards conspiracy.

Furthermore, we define the peer influence probability ${{\rm{PIP}}}_{{\rm{science}}}^{u}$ of ${N}_{s}(u)$ on *u* as the rational number in the range $[0,1]$ given by the complement of $\alpha (u)$ in the positive space of p-values, that is: ${{\rm{PIP}}}_{{\rm{science}}}^{u}=1-\alpha (u)$. Values close to 0 indicate low probability of peer influence, values close to 1 suggest high probability of peer influence. Analogously we define the peer influence probability ${{\rm{PIP}}}_{{\rm{conspir}}}^{u}$ of ${N}_{c}(u)$ on *u*, for any user *u* polarized towards conspiracy.

### Dynamic time warping

Dynamic time warping (DTW) is an algorithm for measuring similarity between two time series *X* and *Y* which computes the optimal (least cumulative distance) alignment between points of *X* (also said query vector) and *Y* (also said reference vector). If *X* has size *n* and *Y* has size *m*, DTW produces an *n* × *m* cost matrix *D* whose $(i,j)$-element is the Euclidean distance d$({\bar{X}}_{i},{\bar{Y}}_{j})$ where ${\bar{X}}_{i}$ and ${\bar{Y}}_{j}$ are obtained by stretching in time the vectors $(X[1],\ldots ,X[i])$ and $(Y[1],\ldots ,Y[j])$ to optimize the best alignment. The value $D(n,m)$ - i.e., the DTW distance between *X* and *Y*, is returned^37^.

We use DTW distance for measuring the similarity between $t(u)$ and $t({N}_{s}(u))$ ($t({N}_{c}(u))$) for any user *u* polarized towards science (conspiracy).

## Results and Discussion

### Anatomy of science and conspiracy pages

**Figure 1:**
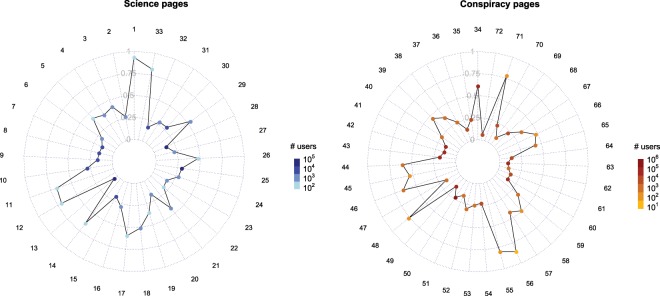
Distribution of likes and likers across the community pages. Plots shows the ratio likers/likes for any Science (left panel) and Conspiracy (right panel) page. Points are colored according to the number of users who liked contents published by the corresponding page (See Tables 2 and 3 for the list of scientific and conspiracy news sources, respectively).

To ensure the robustness of our analysis about the online behavior of polarized users (i.e., if likes are not trivially distributed across pages and if data respect the assumptions of the tests described in *Methods*), we verify the eligibility of the space of our investigation. Namely we study how likers and their activity are distributed over pages and how pages’ activity is distributed over time. Figure 1 shows the distribution of likes and likers across scientific and conspiracy news sources, respectively. Plots shows the ratio likers/likes for every science (left panel) and conspiracy (right panel) page. Points are colored according to the number of users who liked contents published by the corresponding page (See Tables 2 and 3 for the list of scientific and conspiracy news sources, respectively).

Points are mostly localized near the center of the radar chart and, in general, represent the pages with more likers (and more likes). Moreover, points far from the center correspond to pages with the lowest number of likers and likes. This ensures that a comparison between the normalized distributions of likes of two like-minded users (or groups of users) across the community pages is an unbiased estimator of their behavioral difference in terms of liking activity.

**Figure 2:**
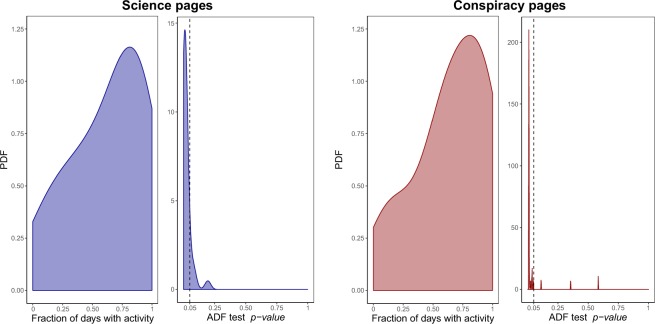
The most pages are active with a nearly constant number of posts almost every day of their lifetime, both in science and conspiracy community. Plots show the PDF of the fraction of days with activity per page and the PDF of p-values obtained by performing ADF stationarity test for all the pages, both in science (left panel) and conspiracy (right panel) community.

Furthermore, in order to investigate how scientific and conspiracy news sources distribute their posting activity over time, we compute the fraction of days with activity of any page with respect to its lifetime - i.e., the temporal distance between its first and its last post. Then we perform an augmented Dickey–Fuller (ADF) test for testing the null hypothesis that a unit root is present in the time series given by the number of posts per day published by a community page during its lifetime. The alternative hypothesis is stationarity (see *Methods* for further details). Figure 2 shows the PDF of the fraction of days with activity per page and the PDF of p-values obtained by performing ADF test for all the pages of science community (left panel) and all the pages of conspiracy community (right panel), respectively.

Plots indicate that the most pages of both the communities are active with a nearly constant number of posts almost every day of their lifetime.

### Experiencing the confirmation bias: polarization and homophily

**Figure 3:**
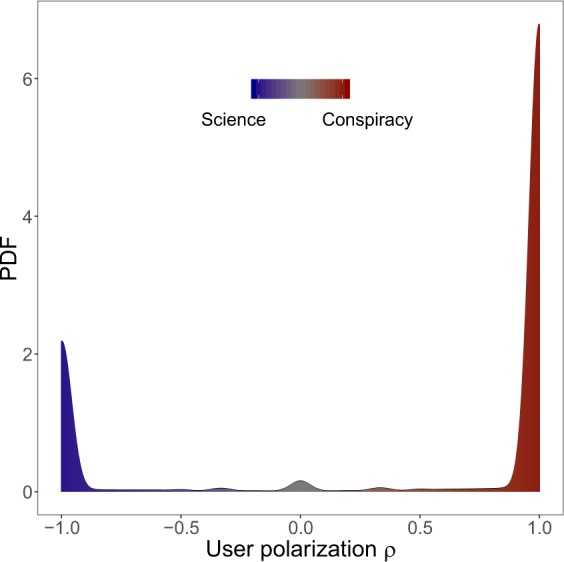
Polarization on contents. PDF of the frequency that a user has polarization $\rho$ is remarkably concentrated in two peaks near the values $\rho =-\,1$ (science) and $\rho =1$ (conspiracy). This indicates that users are clearly split into two distinct communities.

Users’ liking activity across contents of the different categories^2,3,26^ may be intended as the preferential attitude towards the one or the other type of information (documented or not). In Fig. 3 we show that the probability density function (PDF) for the polarization of all the users in *V*^*L*^ is a sharply peaked bimodal where the vast majority of users are polarized either towards science ($\rho (u)\sim -\,1$) or conspiracy ($\rho (u)\sim 1$). Hence, Fig. 3 shows that most of likers can be divided into two groups of users, those polarized towards science and those polarized towards conspiracy. To better define the properties of these groups, we define the set ${V}_{{\rm{science}}}^{L}$ of users with polarization more than 95% towards science

$${V}_{{\rm{science}}}^{L}=\{u\in {V}^{L};\,\rho (u) < -\,0.95\},$$

and the set ${V}_{{\rm{conspir}}}^{L}$ of users with polarization more than 95% towards conspiracy

$${V}_{{\rm{conspir}}}^{L}=\{u\in {V}^{L};\,\rho (u) > 0.95\};$$

such sets corresponds to the two peaks of the bimodal distribution and show how the most users are highly polarized: $|{V}_{{\rm{science}}}^{L}|=243,977$ and $|{V}_{{\rm{conspir}}}^{L}|=758,673$.

**Figure 4:**
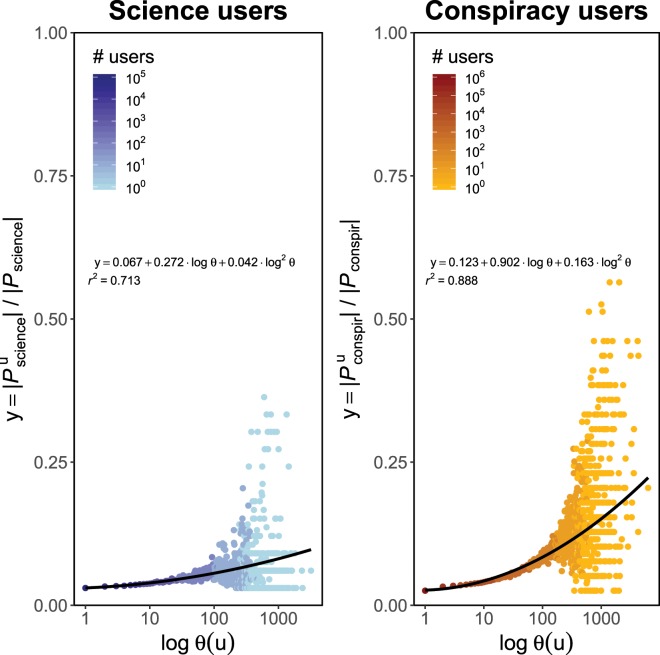
Polarized users span their attention focus on more news sources (and topics) as their engagement grows, but always keeping consistence with their way of thinking. Left panel: users polarized towards science. Right panel: users polarized towards conspiracy. In both panels we plot the average fraction of the total number of community pages where a polarized user *u* distributes her liking activity versus the number of likes log *θ*(*u*) of *u*. Full lines are the results of a quadratic regression model $y={\beta }_{0}+{\beta }_{1}\,\log \,\theta (u)+{\beta }_{2}\,{\log }^{2}\,\theta (u)$, where $y=|{{\mathscr P}}_{{\rm{science}}}^{u}|/|{{\mathscr P}}_{{\rm{science}}}|$ for $u\in {V}_{{\rm{science}}}^{L}$ and $y=|{{\mathscr P}}_{{\rm{conspir}}}^{u}|/|{{\mathscr P}}_{{\rm{conspir}}}|$ for $u\in {V}_{{\rm{conspir}}}^{L}$. Coefficients are estimated using weighted least squares with weights given by the total number of users per engagement value. In both cases, all the p-values are close to zero.

Moreover, for a polarized users $u\in {V}_{{\rm{science}}}^{L}$, in the left panel of Fig. 4, we show the log-linear plot of the average fraction of science pages where *u* is present with liking activity, respect given number of likes *θ* of the user *u*. In the right panel, we show the same quantities for polarized users in ${V}_{{\rm{conspir}}}^{L}$. Figure 4 suggests in both cases a quadratic correlation among the variables; thus, we check whether for a polarized user *u*, the fraction of community pages where *u* spans her liking activity, $y(u)$, can be predicted by means of a quadratic regression model where the explanatory variable is a logarithmic transformation of the number of likes *θ*(*u*), i.e. $y(u)={\beta }_{0}+{\beta }_{1}\,\log \,\theta (u)+{\beta }_{2}\,{\log }^{2}\,\theta (u)$. Using the notation introduced in *Methods*, it is $y(u)=|{{\mathscr P}}_{{\rm{science}}}^{u}|/|{{\mathscr P}}_{{\rm{science}}}|$ for $u\in {V}_{{\rm{science}}}^{L}$ and $y(u)=|{{\mathscr P}}_{{\rm{conspir}}}^{u}|/|{{\mathscr P}}_{{\rm{conspir}}}|$ for $u\in {V}_{{\rm{conspir}}}^{L}$. Coefficients are estimated using weighted least squares with weights given by the total number of users per engagement value and they are – with the corresponding standard errors inside the round brackets - ${\beta }_{0}=0.0669(0.0011)$, ${\beta }_{1}=0.2719(0.0137)$ and ${\beta }_{2}=0.0419(0.0040)$, with ${r}^{2}=0.7133$, for users polarized towards science, and ${\beta }_{0}=0.1229(0.0014)$, ${\beta }_{1}=0.9023(0.0195)$ and ${\beta }_{2}=0.1629(0.0054)$, with ${r}^{2}=0.8876$, for users polarized towards conspiracy. All the p-values are close to zero.

Summarizing, we find that the consumption of polarizing contents is dominated by confirmation bias through the mechanism of challenge avoidance: users polarized towards a narrative tend to consume nearly exclusively content adhering to their system of beliefs, thereby minimizing their cognitive dissonance. Indeed, as their engagement grows, polarized users span their attention focus over a higher number of pages (and topics) keeping consistence with their behavioral attitude.

**Figure 5:**
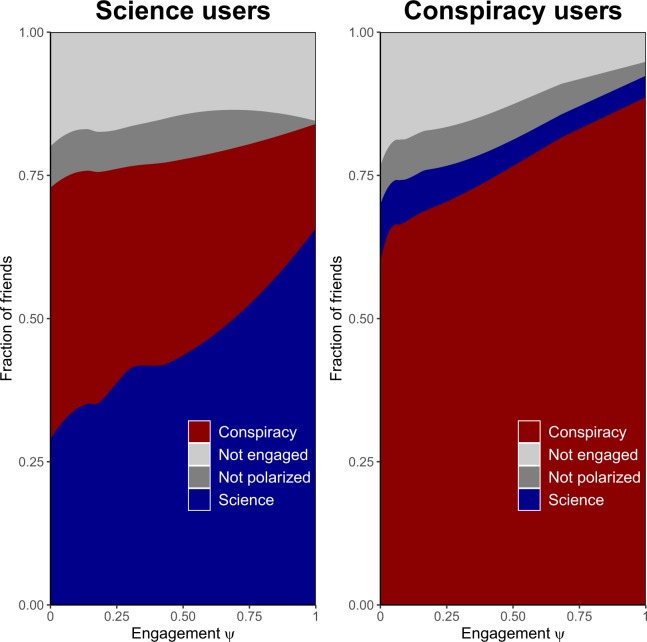
Users not only tend to be very polarized, but they also tend to be linked to users with similar preferences. Fraction of neighbors as a function of the engagement $\psi$. For a polarized science supporter *u*, the fraction of friends *v* with likewise polarization significantly grows with the engagement $\psi$ from $\gtrsim$0.30 to $\gtrsim$0.66. For a polarized conspiracy supporter *u*, the fraction of friends *v* with likewise polarization is very high ($\gtrsim$0.62) and grows with the engagement $\psi$ up to $\gtrsim$0.87 for the most engaged users. A user is labelled as “Not polarized” if she liked the same number of posts from Science pages and Conspiracy pages, respectively. A user is labelled as “Not engaged” if she has no liking activity on the pages of our dataset.

By exploiting the social network of polarized users and their friends, we investigate the role of reinforcement seeking mechanism in the homophily driven choice of friends on Facebook - i.e., the tendency of users to aggregate around common interests. Figure 5 shows the fraction of friends of polarized users as a function of their engagement $\psi (\,\cdot \,)$ both in the case of users in ${V}_{{\rm{science}}}^{L}$ and in the case of users in ${V}_{{\rm{conspir}}}^{L}$. Plots suggest that users not only tend to be very polarized, but they also tend to be linked to users with similar preferences. This is more evident among conspiracists where, for a polarized user *u*, the fraction of friends *v* with likewise polarization is very high ($\gtrsim$0.62) and grows with the engagement $\psi$ up to $\gtrsim$0.87. The neighborhood of a polarized scientific user *u* tends to be more heterogeneous, but the fraction of friends with likewise polarization of *u* grows stronger with the engagement $\psi$ (from $\gtrsim$0.30 up to $\gtrsim$0.66). Furthermore, Fig. 5 clearly indicates that the neighborhood of users engaged with polarizing contents (verified or not) is almost completely polarized as well (74–80% for science users and 72–90% of conspiracy users). The fact that highly polarized users have friends exhibiting an opposite polarization is a direct evidence of the challenge avoidance mechanism: contents promoted by friends which contrast one’s worldview are ignored.

Summarizing, we find that the activity of a user on a polarizing content increases the probability to have friends with similar characteristics. Such information is a precious insight toward the understanding of information diffusion. Indeed, a previous work has shown that users usually exposed to undocumented claims (e.g., conspiracy stories) are the most likely to confuse intentional false information as usual conspiracy stories^3^.

### Engagement, friends and shared news sources

Looking at the self-description of the news sources, several distinct targets emerge both between science pages and between conspiracy pages (see Tables 2 and 3, respectively). This calls for a distinction between friends of a polarized user *u* who share with *u* a similar polarization resulting by liking contents of the same community and friends of *u* who actually like contents promoted by the same pages supported by *u*. In other words, in the first case the user *u* and her neighbourhood are grouped together at community-level (they have same/similar polarization but they like different pages); in the second case the user *u* and her neighbourhood are grouped together at page-level (they like not only pages in the same community but they are also somewhat active on the same set of pages).

**Figure 6:**
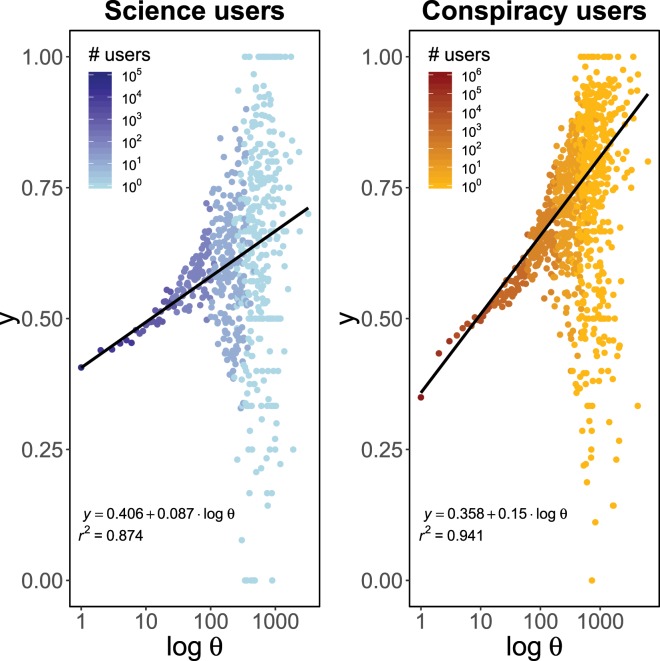
The fractions of science (conspiracy) friends with liking activity on the community pages liked by any given science (conspiracy) user. Left panel: users polarized towards science. Right panel: users polarized towards conspiracy. In both panels, for a polarized user $u$, we plot the average fraction of polarized friends with likewise polarization of $u$ who like contents promoted by the same pages supported by $u$ versus the number of likes log *θ*(*u*) of *u*. Full lines are the results of a linear regression model $y(u)={\beta }_{0}+{\beta }_{1}\,\log \,\theta (u)$. Coefficients are estimated using weighted least squares with weights given by the total number of users per engagement value. In both cases, all the p-values are close to zero.

For a polarized scientific user $u\in {V}_{{\rm{science}}}^{L}$, in the left panel of Fig. 6, we show the log-linear plot of the average fraction *y* of friends $v\in {V}_{{\rm{science}}}^{L}$ with liking activity on the community pages liked by *u*, respect given number of likes *θ* of the user *u*. In the right panel, we show the same quantities for polarized conspiracy users in ${V}_{{\rm{conspir}}}^{L}$. Figure 6 suggests in both cases a linear correlation among the variables; thus, we check whether for a polarized user *u*, the fraction of friends in her category who like contents from the community pages preferred by *u*, $y(u)$, can be predicted by means of a linear regression model where the explanatory variable is a logarithmic transformation of the number of likes $\theta (u)$, i.e. $y(u)={\beta }_{0}+{\beta }_{1}\,\log \,\theta (u)$. Coefficients are estimated using weighted least squares with weights given by the total number of users per engagement value and they are – with the corresponding standard errors inside the round brackets – ${\beta }_{0}=0.4062\,(0.0007)$ and ${\beta }_{1}=0.0869\,(0.0012)$, with ${r}^{2}=0.8744$, for users polarized towards science; ${\beta }_{0}=0.3582\,(0.0007)$ and ${\beta }_{1}=0.1501\,(0.0012)$, with ${r}^{2}=0.9413$, for users polarized towards conspiracy. All the p-values are close to zero. This suggests that polarized users not only tend to surround themselves with friends having similar systems of beliefs, but they actually share with them the involvement within the same community pages.

### Confirmation bias as a filter to peer influence

**Figure 7:**
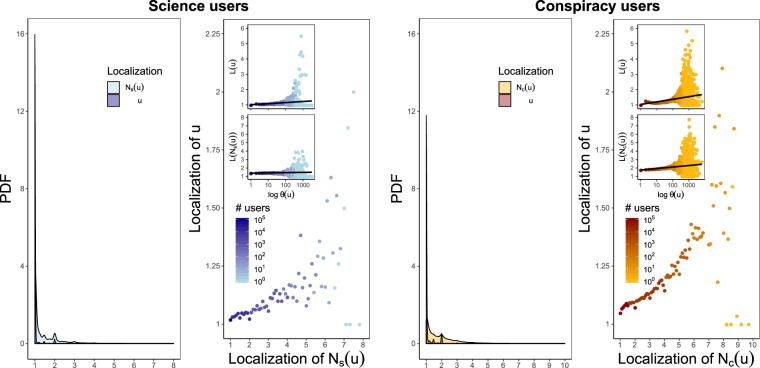
Users tend to remain confined within groups of very few pages even when the corresponding neighborhoods are active on several news sources. For both the communities, plots show the PDF of the localization of a polarized user $u$ and the localization of her polarized neighborhood, and the relationship between these two order parameters. The inset plots show on a logarithmic $x$ scale the relation of $\theta (u)$ with $L(u)$ and $L({N}_{s}(u))\,(L({N}_{c}(u)))$, respectively, for any $u\in {V}_{{\rm{science}}}^{L}$ (${V}_{{\rm{conspir}}}^{L}$). Full lines are the results of a linear regression model whose coefficients are estimated using weighted least squares with weights given by the total number of users per engagement value.

Here we study the liking activity of polarized users in more detail by measuring how they span such activity across the various community pages. For science (conspiracy) community, Fig. 7 shows the probability distribution of the localization *L* along the user set and along the neighborhood set, and the relationship between $L(u)$ and $L({N}_{s}(u))\,(L({N}_{c}(u)))$ for each science (conspiracy) user *u*.

For each polarized user *u*, we observe a positive correlation between these two order parameters: Pearson’s correlation coefficient ${r}_{L(u),L({N}_{s}(u))}\sim 0.5962$ with p-value ~10^−7^ for science community, Pearson’s correlation coefficient ${r}_{L(u),L({N}_{c}(u))}\sim 0.5935$ with p-value ~10^−9^ for conspiracy community. Nevertheless, the most users remain confined within groups of very few pages even with neighborhoods fairly active on several news sources. Moreover, the inset plots of Fig. 7 show on a logarithmic *x* scale the relation of $\theta (u)$ with $L(u)$ and $L({N}_{s}(u))\,(L({N}_{c}(u)))$, respectively, for each $u\in {V}_{{\rm{science}}}^{L}$ (${V}_{{\rm{conspir}}}^{L}$). Full lines are the results of a linear regression model whose coefficients are estimated using weighted least squares with weights given by the total number of users per engagement value.

By investigating the self-description of the news sources, we also find that the most users who decide to span their liking activity over a higher number of pages, choose pages dealing with very interlinked topics ($\gtrsim$76% of science users and $\gtrsim$69% of conspiracy users). Such an evidence suggests that the reinforcement seeking mechanism limits the influence of neighbors and primarily drives the selection and the diffusion of contents even within groups of like-minded people.

### Peer support and reinforcement of preexisting beliefs

So far we have shown how confirmation bias acts as filter to peer influence. In this Section, we investigate the effects of the joint action of confirmation bias and peer influence when the latter does not conflict the cognitive mechanisms of challenge avoidance and reinforcement seeking. Namely, we first compare the liking activity of each polarized user across her preferred community pages with the liking activity expressed on the same pages by the part of her neighborhood with likewise polarization. Then we compare the daily time series given by the number of likes expressed by a polarized user and her like-minded neighborhood, respectively, and we investigate the existence of a causal effect of the latter on the former.

**Figure 8:**
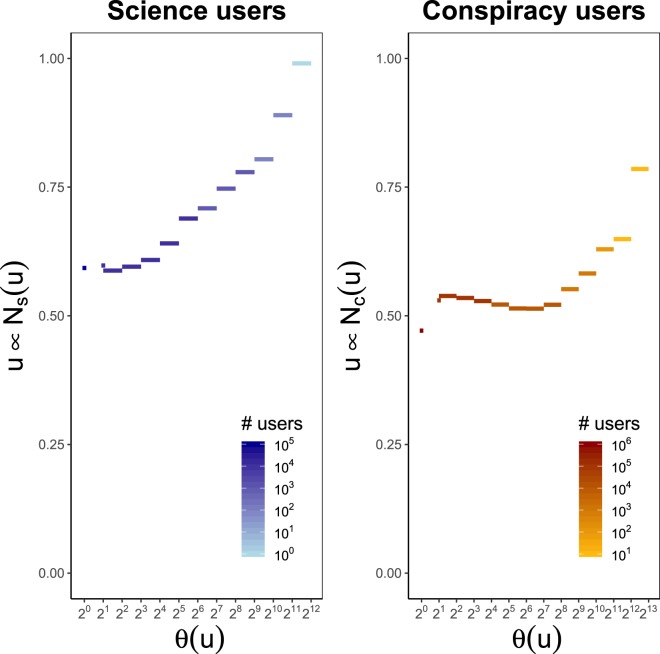
The distribution of likes of a polarized user across her community pages is proportional to the distribution of likes expressed on the same news sources by her neighborhood part with likewise polarization. In the left panel, for any user $u\in {V}_{{\rm{science}}}^{L}$, we show the cosine similarity between the vectors **u** and **N**_**s**_(**u**) whose *k*^th^ component is the number of likes expressed by *u* and ${N}_{s}(u)$ on the *k*^th^ page of ${{\mathscr{P}}}_{science}^{u}$, respectively, versus the number of likes ${\log }_{2}\,(\theta (u))$. Segments represent the average of the cosine measurements regarding users with a liking activity in the range of the corresponding bin. The right panel shows the same quantities for polarized users in ${V}_{{\rm{conspir}}}^{L}$.

For any polarized user $u\in {V}_{{\rm{science}}}^{L}$ we compute the cosine between the versors $\hat{{\bf{u}}}=\frac{{\bf{u}}}{|{\bf{u}}|}$ and $\widehat{{{\bf{N}}}_{{\bf{s}}}}({\bf{u}})=\frac{{{\bf{N}}}_{{\bf{s}}}({\bf{u}})}{|{{\bf{N}}}_{{\bf{s}}}({\bf{u}})|}$ where **u** and **N**_**s**_(**u**) are the vectors whose ${k}^{{\rm{th}}}$ component is the number of likes expressed by *u* and ${N}_{s}(u)$ on the *k*^th^ page of ${{\mathscr P}}_{science}^{u}$, respectively (see *Methods* for further details). The same quantities are calculated for any polarized user $u\in {V}_{{\rm{conspir}}}^{L}$. Figure 8 shows the level of proportionality between the distributions of liking activity of *u* and ${N}_{s}(u)$ (${N}_{c}(u)$) across the pages of ${{\mathscr P}}_{science}^{u}$ (${{\mathscr P}}_{conspir}^{u}$), respectively, versus the number of likes ${\log }_{2}(\theta (u))$ of user *u*. Segments represent the average of the cosine measurements regarding users with a liking activity in the range of the corresponding bin (one of $1,2,(2,4],(4,8],(8,16],\ldots$), and they are colored according to the total number of users belonging to such a range.

The plots show that a polarized user and her likewise polarized neighborhood distribute their likes across her community pages in a similar way, both in science (left panel) and conspiracy (right panel) community. Moreover, except a nearly constant early pattern for conspiracy users, this trend grows with the user engagement suggesting how peer influence acts as a support for reinforcement seeking. Such an interpretation is pointed out more clearly by comparing the temporal evolution of the liking activity of a polarized user and her likewise polarized neighborhood, respectively.

**Figure 9:**
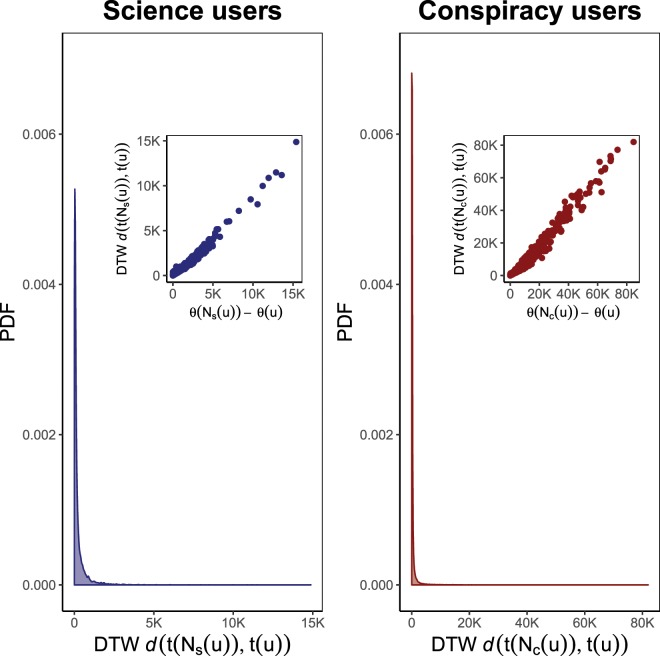
The most users produce a daily time series of likes very similar to the one produced on the same pages by the likes of their respective likewise neighborhood. Left panel: scientific polarized users. Right panel: conspiracy polarized users. PDF of Dynamic time warping (DTW) distance between the daily time series of likes expressed by a polarized user and her likewise polarized neighborhood, respectively. The inset plots show the almost perfect correlation between the difference of liking amount and the corresponding DTW distance, suggesting that extreme DTW distances are due to this factor more than to effective temporal dissimilarity between liking activities.

In order to carry out such an analysis we restrict the observations to those polarized users *u* who exhibit a liking activity large enough to allow the comparison between the time series of likes per day expressed by *u* and her likewise polarized neighborhood, respectively. Namely we define

$${\bar{V}}_{{\rm{science}}}^{L}=\{u\in {V}_{{\rm{science}}}^{L}|\theta (u)\ge {\bar{\theta }}_{{\rm{science}}}\},$$

where ${\bar{\theta }}_{{\rm{science}}}=13$ is the average number of total likes expressed by a user of ${V}_{{\rm{science}}}^{L}$, and

$${\bar{V}}_{{\rm{conspir}}}^{L}=\{u\in {V}_{{\rm{conspir}}}^{L}|\theta (u)\ge {\bar{\theta }}_{{\rm{conspir}}}\},$$

where ${\bar{\theta }}_{{\rm{conspir}}}=12$ is the average number of total likes expressed by a user of ${V}_{{\rm{conspir}}}^{L}$. Furthermore, let $t(u)$ and $t({N}_{s}(u))$ ($t({N}_{c}(u))$) be the time series of likes per day expressed over ${{\mathscr P}}_{{\rm{science}}}^{u}$ (${{\mathscr P}}_{{\rm{conspir}}}^{u}$) by a user $u\in {\bar{V}}_{{\rm{science}}}^{L}$ ($u\in {\bar{V}}_{{\rm{conspir}}}^{L}$) and her likewise polarized neighborhood, respectively. We estimate the temporal similarity between the liking activity of *u* and ${N}_{s}(u)$ (${N}_{c}(u)$) by measuring the DTW distance $d(t({N}_{s}(u)),t(u))$ ($d(t({N}_{c}(u)),t(u))$) (see *Methods* for further details). Figure 9 shows the PDF of such distances for science users (left panel) and conspiracy users (right panel). In both cases we can observe that the most users produce a daily time series of likes very similar to that produced by the likes of their likewise neighborhood. Moreover, the inset plots show the strong positive correlation (Pearson’s coefficient $\gtrsim$0.9887 and $\gtrsim$0.9886 for science and conspiracy, respectively, with both p-values close to zero) between difference in size of *u* liking activity compared to ${N}_{s}(u)$ (${N}_{c}(u)$) and the corresponding DTW distance, suggesting that extreme DTW distances are due to the almost perfect uphill linear relationship more than to an effective temporal dissimilarity between liking activities.

**Figure 10:**
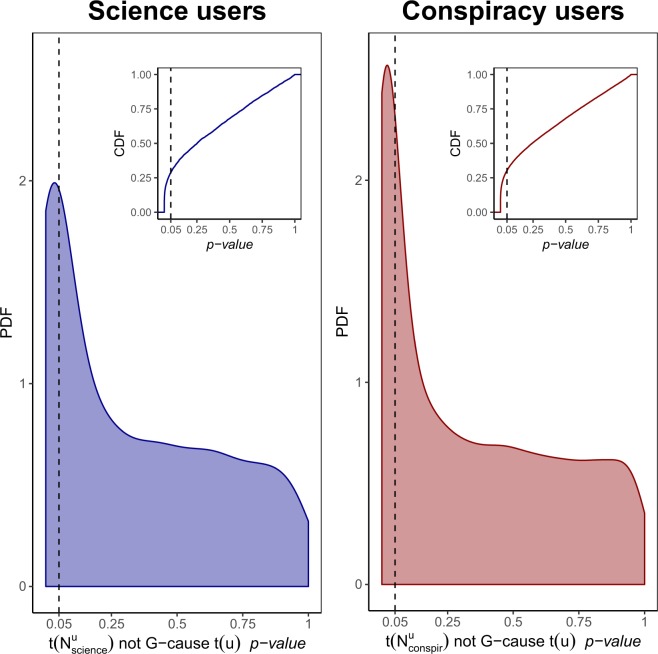
The causal effect of the polarized neighborhood liking activity on the temporal distribution of likes of a polarized user. PDF of p-values of Granger causality tests performed for investigating a causal effect of $t({N}_{s}(u))$ ($t({N}_{c}(u))$) on $t(u)$ for any science user in ${\bar{V}}_{{\rm{science}}}^{L}$ (left panel) and any conspiracy user in ${\bar{V}}_{{\rm{conspir}}}^{L}$ (right panel). The inset plots show the cumulative distribution function (CDF) of the same quantities. Graphics show that the null hypothesis ${{\mathbb{H}}}_{0}$ can be rejected as false.

For each science user in ${\bar{V}}_{{\rm{science}}}^{L}$, we also investigate a causal effect of $t({N}_{s}(u))$ on $t(u)$ by testing the null hypothesis that the former is Granger-noncausal for the latter, namely ${{\mathbb{H}}}_{0}:\,=t{(u)}_{\tau +1}{\mathrel{{\perp\mkern-10mu\perp}}}{ {\mathcal I} }^{\ast }(\tau )|{ {\mathcal I} }_{-t({N}_{s}(u))}^{\ast }(\tau )$. The alternative hypothesis ${{\mathbb{H}}}_{1}$ is predictive causality. The same analysis is repeated for each conspiracy user in ${\bar{V}}_{{\rm{conspir}}}^{L}$ (see *Methods* for further details). In both panels of Fig. 10 we show the PDF of p-values obtained by performing such Granger causality tests. The inset plots show the cumulative distribution function (CDF) of the same quantities. Graphics show that the null hypothesis can be rejected as false: p-values less than the threshold $\bar{\alpha }=0.05$ are more likely than the others in both the communities and represent ~29% and ~34% of the total in science and conspiracy, respectively.

**Figure 11:**
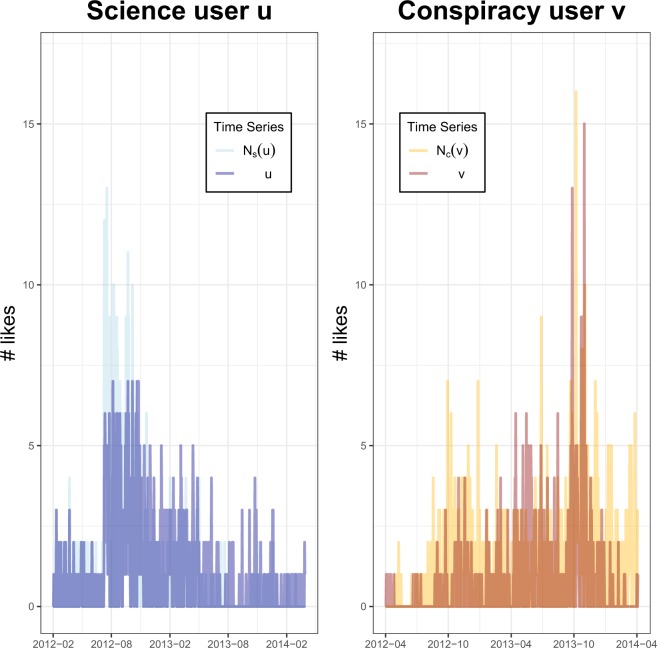
Time series of likes per day expressed by a selected science user *u* and by a selected conspiracy user *v* compared with the daily time series of likes of their neighborhood part *N*_*s*_(*u*) and *N*_*c*_(*v*), respectively. To the pair of time series ($t({N}_{s}(u)),t(u)$) correspondes a DTW distance of 407 and a Granger p-value ~10^−4^ (left panel). To the pair of time series ($t({N}_{c}(v)),t(v)$) correspondes a DTW distance of 463 and a Granger p-value ~10^−5^ (right panel).

As an example, Fig. 11 shows the daily time series of a selected user $u\in {\bar{V}}_{{\rm{science}}}^{L}$ with $\theta (u)=767$ (left panel) and a selected user $v\in {\bar{V}}_{{\rm{conspir}}}^{L}$ with $\theta (v)=488$ (right panel) compared with the daily time series of their neighborhood part ${N}_{s}(u)$ and ${N}_{c}(v)$ who have expressed 779 and 919 likes, respectively. For the pair of time series ($t({N}_{s}(u)),t(u)$), DTW returns a distance equal to 407 and the Granger causality test a p-value ~10^−4^. For the pair of time series ($t({N}_{c}(v)),t(v)$), DTW returns a distance equal to equal to 463 and the Granger causality test a p-value ~10^−5^.

**Figure 12:**
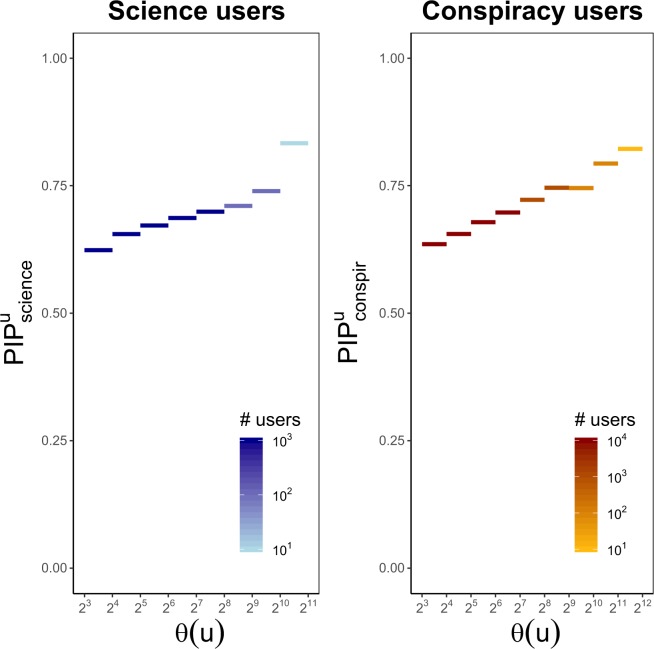
Polarized users reinforce their preexisting beliefs through the influence of their like-minded neighbors. In the left panel, for any user $u\in {V}_{{\rm{science}}}^{L}$, we show the peer influence probability ${{\rm{PIP}}}_{{\rm{science}}}^{u}$ of ${N}_{s}(u)$ on *u* across the pages of ${{\mathscr P}}_{science}^{u}$, versus the number of likes ${\log }_{2}\,(\theta (u))$. Segments represent the average of peer influence probabilities regarding users with a liking activity in the range of the corresponding bin. The right panel shows the same quantities for polarized users in ${V}_{{\rm{conspir}}}^{L}$.

Finally, for each polarized user $u\in {\bar{V}}_{{\rm{science}}}^{L}$, we study the relationship between predictive causality of $t({N}_{s}(u))$ on $t(u)$ and the engagement of *u*. To this aim we use the peer influence probability ${{\rm{PIP}}}_{{\rm{science}}}^{u}$ (see *Methods* for further details) that provides a measure of neighbors influence effectiveness in reinforcing the system of beliefs of *u*. The same analysis is carried out for any polarized user $u\in {\bar{V}}_{{\rm{conspir}}}^{L}$. Figure 12 shows the peer influence probability of *u* versus the number of likes ${\log }_{2}(\theta (u))$ of *u* both in science (left panel) and conspiracy (right panel) community. Segments represent the average of peer influence probabilities regarding users with a liking activity in the range of the corresponding bin, and they are colored according to the total number of users involved in such a range.

Plots show how, in both communities, polarized users reinforce their preexisting beliefs by leveraging the activity of their like-minded neighbors, and this trend grows with the user engagement suggesting how peer influence acts as a support for reinforcement seeking.

## Conclusions

In this paper we studied the effects of confirmation bias experience on the spreading of information in a social network of 1.2 M users engaged with two very distinct and conflicting narratives on Facebook.

Our analyses showed the action of challenge avoidance mechanism in the emergence, around the selected narratives, of two well-separated and polarized groups of users (i.e., echo chambers) who also tend to be surrounded by friends having similar systems of beliefs.

Furthermore, we explored the hypothesis that such a pattern is recursive within a single echo chamber. Despite a shared way of thinking, we proved how during social interactions the strength of confirmation bias is stronger than one could think, leading the action of peer influence into its service and fostering the formation of highly polarized subclusters within the same echo chamber. The fact that polarized users tend to remain confined within groups of very few pages even when the corresponding neighborhoods are active on several news sources, suggests that the reinforcement seeking mechanism limits the influence of neighbors and primarily drives the selection and the diffusion of contents even within groups of like-minded people.

Finally, we investigated the effects of the joint action of confirmation bias and peer influence when this latter does not conflict the cognitive mechanisms of challenge avoidance and reinforcement seeking. Namely, we compared the liking activity of polarized users and the liking activity of their likewise polarized neighborhood, and we test a causal effect of the latter on the former. Our findings revealed that polarized users reinforce their preexisting beliefs by leveraging the activity of their like-minded neighbors, and this trend grows with the user engagement suggesting how peer influence acts as a support for reinforcement seeking.

In such a context, also the positive role played by social influence - e.g., by enabling social learning, seems to lose its effectiveness in the effort to smooth polarization and reduce misinformation risk and its consequences. This makes it even more difficult to design efficient communication strategies to prevent rumors and mistrust.

Internet and social media are the ideal ground for the spread of misinformation to speed up, but individual choices more than algorithms characterise the consumption patterns of users and their friends. Therefore, working towards long-term solutions for these challenges can not be separated from a deep understanding of users’ cognitive determinants behind these phenomena.
