# Author Correction: Recursive patterns in online echo chambers

**DOI:** 10.1038/s41598-021-83758-0

**Published:** 2021-03-09

**Authors:** Emanuele Brugnoli, Matteo Cinelli, Walter Quattrociocchi, Antonio Scala

**Affiliations:** 1 Applico Lab, CNR-ISC, 00185 Rome, Italy; 2 Ca’ Foscari University of Venice, 30123 Venice, Italy; 3 LIMS, the London Institute for Mathematical Sciences, London, UK

Correction to: *Scientific Reports*, 10.1038/s41598-019-56191-7, published online 27 December 2019

The original version of this Article contained an error.

Some of the text in the Introduction was not appropriately attributed. Reference 16 was omitted and is listed below:

Shatz, I. The Confirmation Bias: Why People See What They Want to See. *Effectiviology* https://effectiviology.com/confirmation-bias/ (2018)

As a result, Reference 18 was incorrectly listed as Reference 16 and References 19–38 were incorrectly listed as References 18–37 respectively.

In addition, there was a typographical error. Therefore, the text,

“According to^16^, two primary cognitive mechanisms are used to explain why people experience the confirmation bias:

- Challenge avoidance—i.e., the fact that people do not want to find out that they are wrong,
- Reinforcement seeking—i.e., the fact that people want to find out that they are right.

Though the two are strongly related, and though both behaviors resolve around people’s attempt to minimize their cognitive dissonance—i.e., the psychological stress that people experience when they hold two or more contradictory beliefs simultaneously, challenge avoidance and reinforcement seeking are not inherently linked to each other, and they do not have to occur at the same time.”

now reads:

“As previously described^16^, two primary cognitive mechanisms are used to explain why people experience the confirmation bias^17^:

- Challenge avoidance—i.e., the fact that people do not want to find out that they are wrong,
- Reinforcement seeking—i.e., the fact that people want to find out that they are right.

As has been previously stated^16^, though the two are strongly related, and though both behaviors revolve around people’s attempt to minimize their cognitive dissonance—i.e., the psychological stress that people experience when they hold two or more contradictory beliefs simultaneously, challenge avoidance and reinforcement seeking are not inherently linked to each other, and they do not have to occur at the same time^18^.”

This has now been corrected in the PDF and HTML versions of the Article.

